# Ninth International Medical Congress

**Published:** 1886-11

**Authors:** Richard H. Day

**Affiliations:** P. O. Box 181; Baton Rouge, La.


### NINTH INTERNATIONAL MEDICAL CONGRESS.

### TO THE MEDICAL PROFESSION.

*Dear Doctor :*—In accordance with the request of Dr. Joseph Jones, of New Orleans, La., President of the XIVth Section of the Ninth International Medical Congress, I will investigate the important questions embraced under Section VI of the Inquiries prepared for the Council of this Section by Dr. Jones.

In order to throw light upon these important questions, I have, upon consultation with the President of the XIVth Section, drawn up the following questions, relating chiefly to the effects of overflows and rice-culture upon the Public Health; and, in order, to obtain reliable information, I address the following interrogations to the medical men in those localities, and respectfully request, as early as possible, a full and specific answer to each question.

*Question* 1. How long have you been cognizant of the results of overflows of the Mississippi and its tributaries upon the Public Health ?

2. Have you closely observed the effects of the overflows upon the Public Health?

3. Have you generally or uniformly observed an increase of sickness immediately succeeding the overflows?

4. If so, what diseases? Their character and type?

5. What local or general conditions (including topography) have you noted, controlling or modifying the effects of overflows upon the Public Health?

6. Have you kept statistics of number and dates of these overflows, and the cases of sickness (if any) resulting therefrom? If so, please give these statistics.

7. What is your experience of the effects of rice-culture upon the Public Health? and what the rate of death per 1,000 population before and since the commencement of the rice-culture?

8. Has malarial hæmaturia increased in frequency and severity during the past forty years? And is said increase due to the increased cultivation of rice in the Delta of the Mississippi river, or in other localities where the rice is cultivated?

9. What effect has the camping and working of State prisoners in the low lands and swamps had upon said prisoners, held by Louisiana, Arkansas, Mississippi and other States so employing their prisoners?

10. How many deaths have been caused among State prisoners by malarial diseases, directly traceable to exposure to the malaria of the swamps?

11. Give facts bearing upon the relative effects of overflows and rice-culture upon the colored and white races.

12. Relations of drinking water to the *health* of the inhabitants of rice, sugar, and cotton plantations? Effects of swamp water? effects of cistern, well and spring water, for drinking purposes?

13. The best means of protecting the health of the laborers and inhabitants in such localities?

The physicians of the valley of the Mississippi river and the rice regions of Arkansas, of Alabama, Georgia, North and South

Carolina, Florida and Texas, are earnestly requested to furnish their replies to

RICHARD H. DAY, M. D.,

Baton Rouge, La. P. O. BOX 181.

[We publish the above in the interest of the Congress. In a letter to us, accompanying it, Dr. Day says: "We have been charged in high places with being intellectually dwarfed by *malaria*, and incapable of that mental development which is arrogated to our Northern and Eastern confreres. Let us stamp out this falsehood by the good work which we may present to the Ninth International Congress."

We hope our readers will respond to Dr. Day's call and show that at least in Texas, "Malaria" is not "antagonistic to science."—ED.]

